# Imprinting and expression of *Dio3os* mirrors *Dio3* in rat

**DOI:** 10.3389/fgene.2012.00279

**Published:** 2012-12-06

**Authors:** William H. Dietz, Kevin Masterson, Laura J. Sittig, Eva E. Redei, Laura B. K. Herzing

**Affiliations:** ^1^Program in Human Molecular Genetics, Department of Pediatrics, Children’s Hospital of Chicago Research Center, Feinberg School of Medicine, Northwestern UniversityChicago, IL, USA; ^2^The Asher Center, Department of Psychiatry and Behavioral Sciences, Feinberg School of Medicine, Northwestern UniversityChicago, IL, USA

**Keywords:** imprinting, lncRNA, expression, rat, fetal ethanol exposure, *Dio3*, *Dio3os*

## Abstract

Genomic imprinting, the preferential expression of maternal or paternal alleles of imprinted genes, is often maintained through expression of imprinted long non-coding (lnc) “antisense” RNAs. These may overlap imprinted transcripts, and are expressed from the opposite allele. Previously we have described brain region-specific imprinted expression of the *Dio3* gene in rat, which is preferentially modified by fetal ethanol exposure. The *Dio3os* (opposite strand) transcript is transcribed in opposite orientation to *Dio3* in mouse and human, partially overlaps the *Dio3* promoter, and mirrors total *Dio3* developmental expression levels. Here, we present that the rat *Dio3os* transcript(s) exhibits brain region-specific imprinted expression patterns similar to those of *Dio3*. Rat *Dio3os* transcript expression is also similarly modified by fetal ethanol exposure. Uniquely, both *Dio3* and *Dio3os* expression occur on the same, rather than opposite, alleles, as determined by strand-specific RT-PCR. Future studies will require direct manipulation of the *Dio3os *transcript to determine whether the novel paralleling of total and allele-specific expression patterns of this sense/antisense imprinted gene pair reflects an as-yet undefined regulatory mechanism for lncRNA mediated tissue-specific imprinted expression, or rather is a consequence of a more straightforward, but previously undescribed transcriptional coregulation process.

## INTRODUCTION

Over the past several years, long non-coding RNAs (lncRNAs) have been found to be transcribed across the genome (e.g., Katayama et al., 2005; Huang et al., 2011). Many of these transcripts have a role in the regulation of gene expression, such as via direct overlap with genes or their promoters (reviewed in Amaral and Mattick, 2008) or interaction with and modification of epigenetic chromatin marks (Khalil et al., 2009). Historically, the first lncRNAs were identified within regions of genomic imprinting, characterized by preferential expression of maternal or paternal alleles of imprinted genes. Such imprinted expression is often maintained through expression of “antisense” lncRNAs, which overlap imprinted transcripts and are expressed from the opposite parental allele, potentially “blocking” expression from the “sense” allele on this parental chromosome (reviewed in Peters and Robson, 2008). In addition to direct “antisense” overlap, several imprinted lncRNAs have also been shown to exhibit long-distance *cis* effects on other genes within the regulatory clusters through interaction with chromatin and DNA modifying proteins (Nagano et al., 2008; Pandey et al., 2008), similar to what is being shown for lncRNAs in non-imprinted regions.

The type 3 deiodinase gene, *Dio3*, lies at the distal end of a large cluster of imprinted genes. *Dio3* is preferentially paternally expressed in most tissues (Charalambous and Hernandez, 2012), as are the three other imprinted coding genes in this cluster (Hagan et al., 2009). In contrast, but consistent with the emerging realization of complex allele-preferential gene expression patterns across the genome, we have recently described brain region-specific imprinted expression of *Dio3* in rat (Sittig et al., 2011a). This imprint profile is preferentially modified by fetal ethanol exposure and correlates with behavioral alterations (Sittig et al., 2011b), providing the first evidence of functional consequences of brain region-specific imprinted expression profiles. Although the mechanisms underlying these complex imprint patterns are not yet understood, many possibilities arise through comparison with other imprinted loci, as described above. Immediately adjacent to the *Dio3* gene is a lncRNA transcript, *Dio3os*, that is transcribed in opposite orientation to *Dio3* in mouse and human, partially overlaps the *Dio3* promoter (Hernandez, 2005), and mirrors total *Dio3* developmental and diurnal (Labialle et al., 2008) expression levels. Unlike the large number of large and small non-coding RNA genes within this cluster which are maternally expressed (daRocha et al., 2008), *Dio3os* has been reported to have biallelic, rather than imprinted, expression in murine embryos (Tierling et al., 2006) and in adult mouse cortex (Labialle et al., 2008). To investigate whether the *Dio3os* transcript exists in rat and thus might contribute to complex *Dio3* expression regulation, we have analyzed the total and allele-specific expression patterns of *rDio3os *in naive and ethanol-exposed animals. These analyses demonstrate a combination of features and complexities of *rDio3* expression that together define a novel category within imprinted, lncRNAs.

## RESULTS

### CHARACTERIZATION AND BRAIN REGION-SPECIFIC IMPRINTING OF *rDio3os*

**FIGURE 1:**
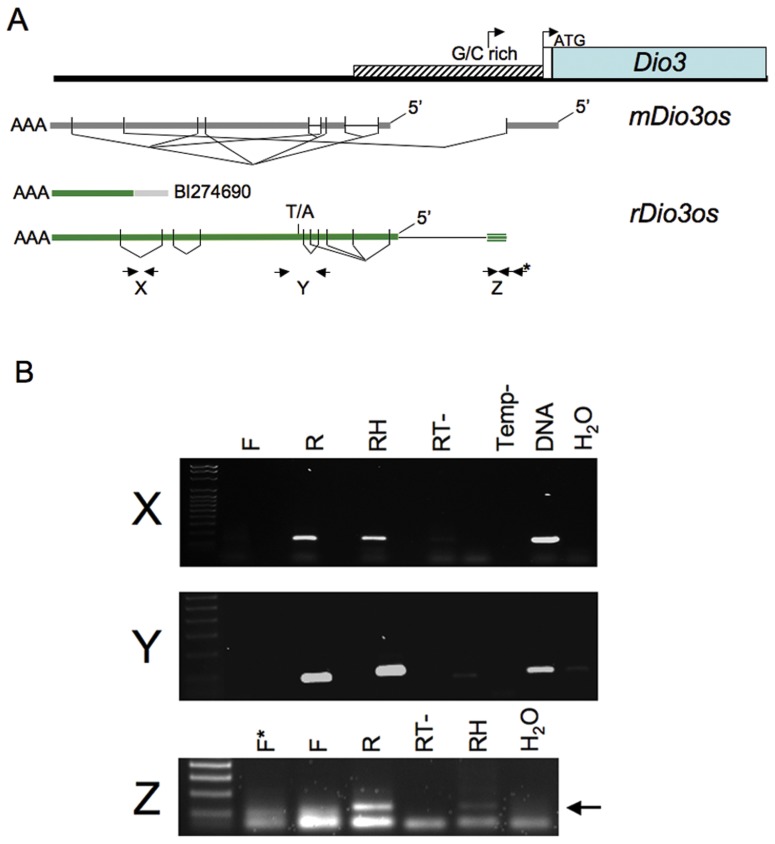
**(A)**Genomic organization of *rDio3os* transcripts across the upstream *rDio3* region. The single *rDio3os* EST along with alternatively spliced RT-PCR and RACE products are shown in comparison to published mouse *Dio3os* exons (Hernandez, 2005) and the 5′ *Dio3* genomic region (Dentice et al., 2007). rDio3 transcriptional (arrow) and translational (ATG) start sites are indicated. Locations of representative primer pairs used for strand-specific RT-PCR (X, Y, Z) are indicated (bold arrows), as is a T/A SNP used for allele-specific expression analysis. **(B)** Representative strand-specific RT-PCR of adult male rat hippocampal (X, Y) or placental (Z) total RNA using primers specific for opposite strand expression (reverse primer: R) or sense expression (forward primer: F) across the *rDio3* upstream region shows expression is derived exclusively from the opposite strand. Controls: *Positive*: Random hexamer-primed RT-PCR (RH), which will copy potential transcripts from either strand; genomic DNA (DNA). *Negative*: Reverse transcriptase deficient (RT^-^) or template deficient (Temp^-^) RT-PCR reactions; template-deficient (H_2_O) PCR reactions.

Transcripts across the upstream *rDio3* region were identified by the single poly-A-containing expressed sequence tag (EST) contained in the NCBI database, and by RT-PCR and rapid amplification of cDNA ends (RACE) across the region from placenta and both neonatal and adult rat frontal cortex (FX) and hippocampus (HP). Both unspliced and alternatively spliced transcripts were identified. *rDio3os *exons overlap those of mouse (Tierling et al., 2006) and extend into the 5′ *rDio3* GC rich region, but splicing and exon–intron boundaries are not identical. Mouse and rat show an average of 83% identity across overlapping regions. Alternatively spliced RT-PCR products are indicated in **Figure 1A**, as are locations of representative primer pairs used for strand-specific RT-PCR (below). We have not been able to identify *rDio3os* splice variants that extend through and overlap the *rDio3* 5′UTR and transcription start site, as has been reported in mouse but not humans (Hernandez et al., 2004), although strand-specific RT-PCR has identified the presence of at least an 154 bp opposite strand (OS) transcript 60 bp upstream of the standard *rDio3* 5′UTR, within the minimal promoter and potentially overlapping an alternative transcription start site identified in keratinocytes (Dentice et al., 2007; **Figure 1B**, “Z”).

### UPSTREAM EXPRESSION IS DERIVED EXCLUSIVELY FROM THE OPPOSITE STRAND TO *rDio3*

The EST BI274690, ~3–4 kb upstream of *rDio3* (inclusive), does not encode an open reading frame but contains a poly-A sequence, strongly suggesting that its direction of transcription is opposite to that of *rDio3* and that it represents the *rDio3os* transcript rather than an extended *rDio3* 5′UTR variant. We identified this poly-A sequence in RACE products as well. However, early reports of* rDio3* expression based on Northern blot hybridization from rat brain (Tu et al., 1999) suggested the presence of alternative, longer (>2.1 kb) *rDio3* transcripts, with later studies in human tissue demonstrating *DIO3* transcription overlapping canonical *DIO3* promoter sequences, as well as identifying a longer, 4.8 kb transcript that hybridized exclusively to the 5′ but not 3′ regions of *DIO3* and was thus suggested not to encode the DIO3 protein (Hernandez et al., 2004). This 4.8 kb transcript would be predicted to overlap the 5′ end of the *mDio3* 5′UTR-overlapping *mDio3os* splice variant (Hernandez et al., 2002) and would be expected to extend at least 2 kb further 5′, depending on splicing profiles, therefore giving it further *Dio3os* overlap potential. However, in these experiments, (double-stranded) cDNA rather than allele-specific probes were utilized, and thus it is possible that the *DIO3* promoter sequence within the extended *DIO3* transcript actually hybridized with *DIO3os* transcripts instead. To confirm that transcription across the 5′ *rDio3* region identified herein in fact occurs exclusively in the opposite orientation to *rDio3*, we performed strand-specific RT-PCR across multiple sites, including X, Y, and Z (**Figure 1A**), in several different tissue regions/developmental time points (e.g., adult HP, **Figure 1B**). As indicated in **Figure 1B**, RT-PCR product was obtained only when primers complementary to the *rDio3os* transcript were used. OS transcription was confirmed for both unspliced (e.g., across the EST BI274690, “X”) and spliced regions (“Y”).

### IMPRINTED *rDio3o*s EXPRESSION ARISES FROM THE SAME ALLELE(S) AS THE ADJACENT *rDio3* TRANSCRIPT

The *rDio3os* transcript had been reported to lack imprinted expression in mouse embryos (Tierling et al., 2006) and in adult mouse cortex (Labialle et al., 2008). However, we have recently discovered that imprinting of the *rDio3* transcript exhibits brain regional, strain, and developmental specificity (Sittig et al., 2011a), with the greatest differentials occurring in fetal FX, where imprinted, paternal expression was observed in reciprocal crosses, relaxing to biallelic expression in adult FX, and in adult HP, where *rDio3* expression was imprinted and derived from the maternal allele in one cross but biallelic in the reciprocal cross. As is customary for antisense transcripts in imprinted loci, we expected that *rDio3os* expression would be oppositely imprinted from *rDio3*, or equally biallelic. To determine whether the *rDio3os* transcript also exhibited complex imprinted expression patterns, we identified single-nucleotide polymorphisms (SNPs) within the *rDio3os* transcribed region between Sprague-Dawley (SD) and Brown-Norway (BN) rat strains. Focusing on a T/A SNP distant from G/C-rich or repetitive sequence regions (**Figure 1A**), we then examined expression across this SNP in animals derived from reciprocal crosses of these strains (S × B or B × S), where the first strain represents the maternal allele. We performed allele-specific expression analysis by strand-specific RT-PCR followed by direct sequencing on dissected frontal cortices and hippocampi from fetal and adult animals. As our prior experiments determined that expression across the region derived exclusively from the OS direction, we also performed direct sequencing on products isolated following random hexamer (RH)-primed RT-PCR reactions from these regions, as well as across several SNPs in placenta.

**FIGURE 2:**
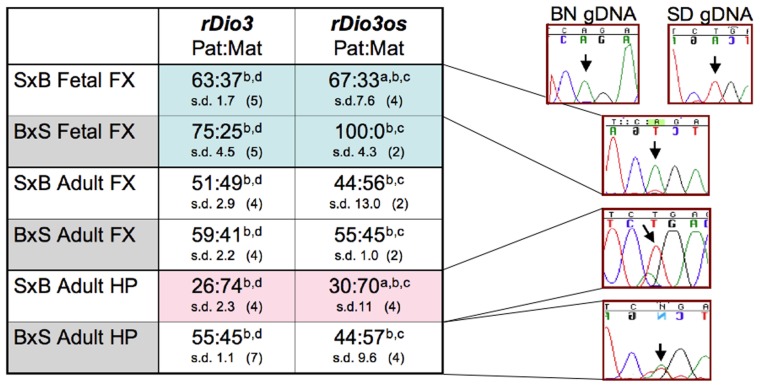
**Allele-specific expression analysis demonstrates that *rDio3os* expression is imprinted in a strain, region, and developmental-specific manner paralleling that of *rDio3*.** Here, various imprinted expression patterns are shown for fetal and adult male hippocampus (HP) and frontal cortex (FX), respectively. *rDio3* imprinted expression is reproduced from our previous work (Sittig et al., 2011a). Relative *rDio3os* expression across a SNP between Sprague-Dawley (SD; T allele) and Brown-Norway (BN; A allele) animals was determined following (a) strand-specific or (b) random hexamer-primed RT-PCR, followed by (c) direct sequencing or (d) pyrosequencing analysis (Sittig et al., 2011a). For each cross, the maternal strain is given first (S × B or B × S). For allele-specific expression, paternal:maternal (Pat:Mat) expression ratios are given, along with representative preferential paternal, maternal or biallelic sequence traces. Strand-specific allele-specific analysis of *rDio3os* samples performed in duplicate-quadruplicate. s.d., standard deviation.

As shown in **Figure 2**, *rDio3os* expression in brain is imprinted in a strain, region, and developmental-specific manner paralleling that of *rDio3* (Sittig et al., 2011a). Specifically, both *rDio3* and *rDio3os* show relatively biallelic expression patterns in adult FX from both S × B and B × S crosses, comparable to the biallelic expression reported in adult mouse cortex (Labialle et al., 2008). In the adult HP from the B × S cross, both *rDio3* (Sittig et al., 2011a) and *rDio3os* are also biallelic. In placenta, the predominant, unspliced *rDio3os* transcripts, which comprise ~80% of total *rDio3os*, are also biallelically expressed (58:42 s.d. 5.6, *n* = 3), consistent with what had been observed for *Dio3* in rat (Sittig et al., 2011a) and mouse (Yevtodlyenko et al., 2002), although others have reported preferential paternal *mDio3* expression (Tsai et al., 2002).

Surprisingly, however, not only is *rDio3os* imprinted in both S × B and B × S fetal FX, and in S × B adult HP as is *rDio3* (Sittig et al., 2011a), but the expression arises from the *same allele* as that of *rDio3* (**Figure 2**). Analysis of the spliced placental *rDio3os* transcripts suggest that these, also, exhibit imprinted expression, redolent of the conflicted status of placental murine *Dio3* expression. The more common (“Y”) variant demonstrates preferential paternal expression (86:14 s.d. 20, *n* = 3; *p* > 0.05 compared to unspliced biallelic transcript), whereas a minor alternative splice variant exhibits exclusively maternal expression in two of three placenti. In placenta, we also observe unspliced biallelic and spliced paternal expression across a downstream SNP, although its location distant from splice junctions and adjacent to a region of repetitive element sequence homology precluded similar examination in brain samples, which have markedly lower* rDio3os* expression levels than does placenta. Thus, unlike the typical sense/antisense arrangement in imprinted genetic loci, where the antisense transcript arises from the opposite allele from that of the sense transcript, in brain and potentially in placenta, both *rDio3* and *rDio3os* transcripts arise from the same allele.

### TOTAL *rDio3os* EXPRESSION Tracks *rDio3*: STRAIN AND BRAIN REGION-SPECIFIC EFFECTS

The standard model for sense and antisense expression in imprinted genomic loci is that, in general, expression of the antisense transcript occludes expression of sense transcripts, through direct overlap (Williamson et al., 2011) or *in trans* as a negative regulator of expression through interaction with complexes at the gene promoter (Shin et al., 2008). Expression of such sense and antisense genes is thus usually complementary, or inversely proportional. In contrast, most lncRNAs located near coding genes have been found to independently transcribed (Liao et al., 2011). Of the ~2% coregulated with coding genes, most are not found in the “head-to-head” orientation of the *Dio3/Dio3os* transcripts (Mercer et al., 2008; Liao et al., 2011), as currently characterized (Hernandez et al., 2004). As *Dio3os* total expression has been reported to be roughly correlated with *Dio3* *in vivo* during development, adulthood (Hernandez et al., 2002; Labialle et al., 2008), and in tissue culture (Kester et al., 2006) for mouse and human, respectively, we wanted to determine whether this coregulation extended to the regional and strain-specific total expression pattern observed for *rDio3* in brain. Furthermore, we aimed to determine if *rDio3os* exhibited transcriptional response to fetal alcohol exposure similar to that of *rDio3* (Sittig et al., 2011b), despite its novel imprinted, homo-allelic expression profile and unusual genomic organization.

**FIGURE 3:**
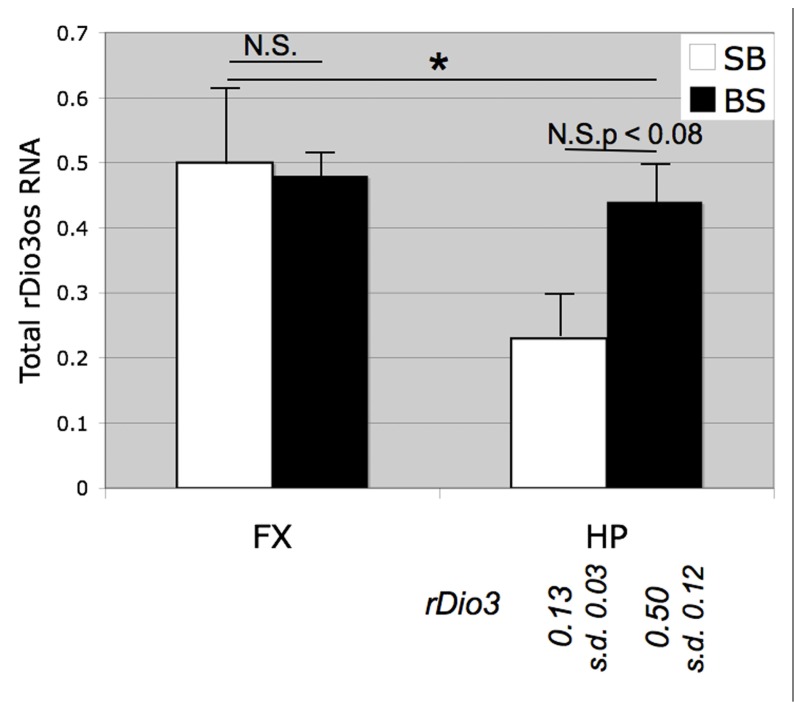
**Strain and brain region-specific effects on total *rDio3os* expression.** As for *rDio3*, semi-quantitative RT-PCR from adult male cortex demonstrates no significant difference between animals derived from a B × S cross vs. an S × B cross (*p* > 0.84), whereas animals from the S × B cross exhibit significantly decreased total *rDio3os* expression in the hippocampus vs. cortex. S × B adult hippocampi also trend toward decreased *rDio3os* expression as compared with hippocampi from the B × S cross (*p* > 0.08). For comparison, total *rDio3* adult male hippocampal RNA expression is indicated below bars (from Sittig et al., 2011a; *n* = 7–8). Samples are normalized to rat beta-actin expression levels. *n* = 3–4; extreme outliers omitted from analysis. *p > 0.05; N.S., not significant; HP, hippocampus; FX, frontal cortex; s.d., standard deviation.

Initially, we examined total *rDio3os* expression from micro-dissected rat brain regions (**Figure 3**). We found that despite the low levels of expression of this transcript we were able to achieve reliable amplification using concentrated cDNA samples in a semi-quantitative RT-PCR assay, whereas we were unable to generate useable standard curves or reproducible relative expression profiles for *rDio3os* using real-time qRT-PCR amplification cocktails. Previously, we had found that in the adult male S × B rat brain, total *rDio3* mRNA expression levels are lower in the HP than in the FX, but these regions exhibit similar total *rDio3* expression levels in the B × S cross (Sittig et al., 2011a). We confirmed a similar pattern for *rDio3os*, wherein *rDio3os* expression is significantly reduced in the male adult HP compared with that in the FX in the S × B cross, but is statistically indistinct between regions in the B × S cross.

**FIGURE 4:**
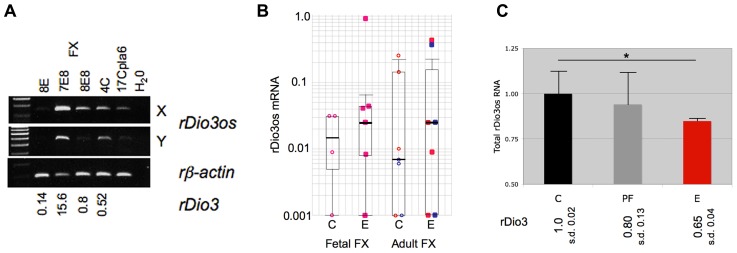
**Coordinate response of *rDio3* and *rDio3os* to prenatal ethanol exposure.** **(A)** Semi-quantitative RT-PCR from fetal frontal cortex of individual control (C) and ethanol-treated animals (E) demonstrates levels of *rDio3os* expression tracking total *rDio3* expression (relative levels from qRT-PCR in triplicate, normalized to beta-actin; L.S.) for both unspliced (X) and potentially spliced (Y) transcript-specific primer pairs. All animals are from an S × B cross. A placental *rDio3os* sample shown for comparison. Note that 4C is an upper outlier from controls to allow visualization of expression product. **(B)** Semi-quantitative RT-PCR from female fetal or adult rat S × B frontal cortex (FC) from control (C) or prenatally ethanol-exposed animals (E). Pink/red: female; blue: male. **(C)** Semi-quantitative RT-PCR from adult male hippocampus from control (C), pair-fed (PF), and prenatally ethanol-exposed animals (E) demonstrates decreased total *rDio3os* expression (region Y, shown) in ethanol-exposed animals. Relative rDio3 protein levels (from Sittig et al., 2011b; *n* = 4–6) are given below each category. As in fetal brain, the direction of change is the same as that for *rDio3* although the absolute degree of change is less. Samples run in duplicate, normalized to beta-actin; outliers from each three sample group excluded. *p > 0.05, Student’s *t*-test. s.d., standard deviation.

We then observed that, as with native expression, total *rDio3os* response to prenatal ethanol exposure mirrors *rDio3* expression. **Figure 4A** illustrates RT-PCR products from representative individual S × B fetal brain regions, demonstrating that expression across the *rDio3os* transcript roughly correlates with relative *rDio3* expression for that individual, as measured by real-time qRT-PCR. Overall, we observed elevated *rDio3os* expression in ethanol-exposed (E) vs. control (C) FX in three of six female fetal samples, and in three of seven adult samples, by gender (**Figure 4B**), consistent with the overall increase in *rDio3* expression observed in ethanol-exposed FC from these respective groups (Sittig et al., 2011b). The exponential increase in *rDio3os* expression between E and C groups together and separately by developmental stage is highly significant by Chi-square analysis (*p* > 0.0001) although the absolute differences in mRNA expression levels between groups are not significant by two-way ANOVA. In contrast, *rDio3os* expression in adult male HP was decreased following ethanol exposure (**Figure 4C**), again consistent with the pattern observed for *rDio3* (Sittig et al., 2011b). Together, these results demonstrate that total *rDio3os* expression patterns are regulated in a similar manner to those of *rDio3*, albeit with somewhat greater variability and to a somewhat lesser degree than the coding transcripts.

## DISCUSSION

The present study demonstrates the coordinate expression and imprinting profile of a non-overlapping lncRNA/gene pair. Specifically, we show that the rat *Dio3os* transcript does not obviously overlap the *rDio3* transcript itself, that it is imprinted, and that it is coregulated with *rDio3* both at the level of total expression and of imprinted expression. To our knowledge, this represents the first example of paired sense/OS transcripts arising from the same allele. The original paradigm for (long) non-coding RNAs (lncRNAs) was that they were usually found in imprinted gene clusters, overlapped at least one imprinted coding transcript (in antisense orientation), and were themselves reciprocally imprinted to the protein-coding gene (O’Neill, 2005; Peters and Robson, 2008). Subsequent identification of roles for imprinted lncRNAs in the regulation of imprinted expression of non-overlapping genes opened our eyes to the importance of these transcripts as direct functional entities (Nagano et al., 2008; Pandey et al., 2008). In turn, recently published global analyses of lncRNAs has highlighted many non-overlapping lncRNA/gene pairs that frequently show coordinate expression profiles (Mercer et al., 2008; Ponjavic et al., 2009; Liao et al., 2011), with lncRNAs implicated in some cases in regulation of expression of the paired gene (Khalil et al., 2009; Ponjavic et al., 2009). Here, we have shown that, much like *Archaeopteryx*, the rat *Dio3os* transcript exemplifies both of these paradigms, carrying features of and serving as a span between the broad and generalized class of lncRNAs, and the more specialized category of imprinted non-coding transcripts.

The great majority of lncRNAs that have been identified within imprinted gene clusters are themselves imprinted, or have tissue-specific imprinted isoforms (Numata et al., 2010), and thus our observation of region-specific imprinting of *rDio3os* (**Figure 2**), previously reported to exhibit biallelic expression, might not be considered particularly unusual. Slightly more unusual is the genomic organization of *Dio3/Dio3os* (**Figure 1**), which at face value appears to be a bidirectional, head-to-head pairing of sense (coding) and antisense, or OS, transcripts, with no detectable sequence overlap in rat (this manuscript), or human (Hernandez et al., 2004), although a small 5′ overlap has been identified in mouse (Hernandez et al., 2002). This is in contrast to the multiple sense/antisense pairs of coding/non-coding RNAs with significant overlap found within imprinted gene clusters (Katayama et al., 2005; O’Neill, 2005). Whereas there are also many imprinted lncRNAs within these clusters that do not overlap coding transcripts (e.g., Zhang et al., 2011) and which may technically be in antisense orientation to coding genes, including ncRNAs with functional roles on non-overlapping transcripts such as *Air* (Nagano et al., 2008) and *Kcnq1ot1* (Pandey et al., 2008), the distance between these is usually much larger than that between the identified *Dio3os* and *Dio3* transcripts. There does exist a possibility of transcriptional overlap between *Dio3os* and *Dio3*, as Hernandez et al. (2004) have identified a putative *Dio3os* promoter downstream of *Dio3*, which could facilitate chromatin reorganization across the *Dio3* locus. In addition, as neither we (*rDio3os*) nor others (Hernandez, 2005) have been able to identify the absolute 5′ end of the *Dio3os* transcript using RACE or other methods, there also remains a likelihood that the full *Dio3os* transcript flanks the *Dio3* gene itself, such that transcription proceeds across *Dio3* without overlap in the final processed products. In either case, this organizational structure is highly unusual for imprinted as well as non-imprinted lncRNA/coding RNA gene pairs (Ponjavic et al., 2009), most especially when the latter show evidence of coregulation (Mercer et al., 2008; Liao et al., 2011), as do *Dio3/Dio3os* (Hernandez et al., 2002; **Figures 3 and 4**).

Most unusual is our finding that imprinted *rDio3os* expression originates from the same allele as that of *rDio3*. Within imprinted clusters, lncRNAs are almost universally transcribed from the allele opposite to the imprinted coding transcripts in the regulatory domains (O’Neill, 2005). To our knowledge, *rDio3/rDio3os* represent a previously unreported and novel scenario, wherein both an imprinted gene and its immediately adjacent non-coding RNA are transcribed from the same, rather than opposite, parental alleles. The closest comparison to this scenario occurs within the complex *Gnas* locus, where both *Nespas* (overlapping *Nesp55*) and *Gnasxl* (one of several alternative transcripts of *Gnas*, along with *Nesp55*) are paternally expressed, in opposite orientation (Williamson et al., 2006), albeit further apart than *Dio3/Dio3os* (Williamson et al., 2002). However, unlike *Dio3*/*Dio3os*, the expression of *Nespas* and *Gnasxl* is not concordant across tissues, although both are expressed in heart and brain (Pasolli et al., 2000; Wroe et al., 2000; Plagge et al., 2004). Furthermore, the *Dio3* promoter has not been found to exhibit differential methylation (Tsai et al., 2002), and, as mentioned above, may not itself drive *Dio3os* expression (Hernandez et al., 2002), whereas expression of *Nespas* and *Gnasxl* is regulated by a differentially methylated region (DMR) between them, that controls imprinted expression across the locus (Williamson et al., 2006). Finally, although the imprint of both pairs is maintained by a distant DMR, for *Gnasxl* this is downstream of *Nespas* (Frohlich et al., 2010), whereas imprinted *mDio3* expression is regulated by a DMR almost 1 Mb distant (Lin et al., 2003).

This novel collection of features suggests that the regulation of expression of *Dio3/Dio3os* likely differs from the standard paradigm for coding/non-coding genes within imprinted loci, and may be better represented by that observed for coregulated coding/lncRNA pairs. In imprinted loci, expression of the non-coding transcript generally forestalls expression of the coding gene on that allele, either by direct transcriptional (stochastic) interference, often leading to epigenetic changes in the chromatin of overlapped regulatory regions (Williamson et al., 2011), or by recruitment of chromatin modifying factors to (Pandey et al., 2008) or direct interaction with (Nagano et al., 2008) the promoters of neighboring genes. In comparison, about 20% of non-imprinted lncRNAs are coregulated with their neighboring coding gene when in a sense/antisense orientation (Katayama et al., 2005), although coding/lncRNA pairs in all orientations have been described that are coordinately, inversely, or randomly regulated (Mercer et al., 2008). Of course, coregulation of adjacent genes may simply reflect a similar chromatin environment, and the unique expression profile of *rDio3/rDio3os* may not imply a regulatory role for either in generating that profile. On the other hand, lncRNA members of coregulated gene pairs have, in many instances, been shown to exhibit regulatory function on the coding gene (Katayama et al., 2005; Mondal et al., 2010). They have also, for example, been identified as chromatin-associated RNAs (CARs; Mondal et al., 2010), that positively regulate transcription of neighboring genes via establishing active chromatin structures through interaction with the chromatin. In that study, however, *Dio3os* was not identified among the CARs, although another ncRNA in the *Dlk1-Dio3* locus, *Meg3*, which exhibits standard opposite imprint expression to *Dio3*, was. Tellingly, neither has *Dio3os* been pulled out in several other large-scale studies on lncRNAs, including those identified as associated with chromatin modifying factors (which tend to decrease expression of adjacent genes; Khalil et al., 2009), those identified by evolutionary sequence constraints (Ponjavic et al., 2009), chromatin state (Guttman et al., 2009), expression in brain (Mercer et al., 2008) or across transcriptome analysis (Katayama et al., 2005), and micro-array platforms (Liao et al., 2011). In addition, *Dio3os*, despite being characterized in the published literature (Hernandez et al., 2004) and listed in lncRNAdb and NONCODE, databases of non-coding RNAs (Amaral et al., 2011; Bu et al., 2012), contains no expression profiling information in these, nor in the Allen Brain Atlas (Lein et al., 2007) and its low level and tissue-specific expression may preclude it from being identified in such screens.

Despite the apparently elusive nature of the *Dio3/Dio3os* partnership in large-scale screens for lncRNA functionality, the conservation of sequence along with coregulation of total expression between species and maintenance of tissue-specific imprint patterns in wild-type and developmentally substandard conditions described herein, suggest that coregulation is actively maintained and retains the possibility of a role for *Dio3os* in active regulation of imprinted *Dio3* expression. As a coregulated gene pair, *Dio3os* might enhance or, contrarily, temper expression of *Dio3*, both scenarios having been observed for coregulated lncRNA/coding gene pairs (Katayama et al., 2005; Mondal et al., 2010; Vance et al., 2011). Multiple potential mechanisms exist for these possibilities, similar to those observed for other lncRNA-based regulation. Whether the *Dio3os* transcript originates within the *Dio3* promoter region or from the flanking, 3′ end of *Dio3*, active *Dio3os* transcription across the locus may create an “open” chromatin structure permissive for transcriptional machinery action upon the *Dio3* gene. Alternatively, the resultant chromatin structure may limit accessibility, tempering but not occluding *Dio3* transcription, as would be expected from an imprinted antisense transcript expressed from the allele opposite to that of gene expression. Direct transcriptional overlap might also regulate the post-transcriptional stability of *Dio3* by dsRNA or siRNA formation via pre-processed *Dio3os* transcripts spanning *Dio3*, or an as-yet-unidentified *Dio3os* 5′ exon within the *Dio3* 3′UTR (Hernandez, 2005). Active *Dio3os* transcription and presence of the *Dio3os* transcript itself adjacent to the *Dio3* promoter, or overlapping it (Hernandez et al., 2002; Dentice et al., 2007), may prevent binding of an inhibitor protein, thus serving to activate *Dio3* expression, or limit binding of a transcription factor or Pol II recruitment, thus moderating expression. Another possibility is that, as for several other lncRNAs, the *Dio3os* RNA itself may be functional, homologously pairing directly with the *Dio3* promoter region and recruiting activating or inhibitory factors, or regionally interacting with chromatin modification factors to epigenetically alter the chromatin landscape, facilitating or tempering *Dio3* expression. Any of these models offer the format of an additional layer of transcriptional regulatory control for *Dio3*, which, while possibly being an evolutionary relic, may in fact be necessary to effect proper control of total *Dio3* expression levels, and thus of circulating thyroid hormone levels in the developing and adult animal, in a temporal and regional-specific manner.

These studies demonstrate that the *Dio3os* transcript is present in rat, and that its structure is generally conserved with that in mouse. As in mouse, total *rDio3os* levels generally track those of *rDio3*. Our detailed analyses further demonstrate that total expression similarities for the *rDio3* gene and the *rDio3os* lncRNA are maintained and are similarly influenced by developmental stage, brain region, strain background, and prenatal insult (ethanol exposure).

We further demonstrate that *rDio3os* expression is also imprinted, as is common for lncRNAs within imprinted clusters. As for total expression, imprinted *rDio3os* expression patterns vary in line with those of *rDio3*. Although this could simply be a situation of coregulation of these genes, it is tempting to speculate that *rDio3os* expression may be positively regulating *rDio3* expression, as has been described for other non-imprinted lncRNAs/-adjacent genes (Katayama et al., 2005; Mercer et al., 2008; Mondal et al., 2010).

Future studies including identification of *rDio3os* transcriptional overlap of *rDio3* regulatory regions, as in mouse and humans, and knockdown and truncation of the *Dio3os* transcript in neuronal tissue will be required to evaluate the requirement of *Dio3os* expression in *Dio3* transcriptional regulation. In comparison with what is known for other lncRNA/coding gene pairs, the direction of *Dio3os*-mediated regulation of *Dio3*, if any, will guide further studies addressing the highly unusual mono-allelic expression of these genes and the mechanism of their coregulation or sequential regulation. In turn, understanding these mechanisms may aid in addressing malfunctions in *Dio3* expression patterns, among others, and their phenotypic consequences, such as are observed following prenatal ethanol exposure (Sittig et al., 2011b).

## MATERIALS AND METHODS

### ANIMALS

Animal procedures and tissue collection and processing were as described (Sittig et al., 2011a,b) Animal procedures were approved by the Northwestern University Animal Care and Use Committee. Adult SD and BN male and female rats (70–85 days of age, Harlan, Indianapolis, IN, USA) were mated (*n* = 6 SD females, *n* = 8 BN females) to obtain reciprocal F1 hybrid offspring, B × S and S × B, with first letter of the maternal strain first followed by the first letter of the paternal strain. For the fetal study, dams were sacrificed by decapitation on gestational day 21 (G21). Additional pregnant dams (BN *n* = 7; SD *n* = 8) were allowed to give birth and rear litters. One or two males and one or two females from each litter were sacrificed following behavioral testing (Sittig et al., 2011a,b) after 60 days of age. Prenatal ethanol (E) exposure and maternal diet procedures were performed as described previously (Sittig et al., 2011b). Briefly, pregnant females were assigned to a diet group on G8: E dams received an ethanol-containing (5% w/v, 35% ethanol-derived calories) liquid diet between G8 and G21 (Lieber-DeCarli ’82; Bio-Serv, Frenchtown, NJ, USA). Pair-fed (PF) dams received an amount of isocaloric liquid diet (Lieber-DeCarli ’82; Bio-Serv, Frenchtown, NJ, USA) that matched the paired E dam’s diet consumption on the previous day. Liquid diets were replaced with lab chow on G21, while control (C) dams received lab chow and water *ad libitum* from G1 to G21.

### TISSUE COLLECTION

Pregnant dams were sacrificed by decapitation on G21 between 1000 and 1200 hours, as previously described (Sittig et al., 2011a,b). Fetal heads were collected directly into RNA*later* reagent (Ambion, Austin, TX, USA) and kept at room temperature for 24 h before brain regions were dissected and transferred into fresh RNA*later*. Fetal frontal cortices were dissected using a microscope according to the *Atlas of Prenatal Rat Brain Development*: A/P (1.0–2.2; M/L 0.0–1.5; D/V 0.0–1.0; Altman and Bayer, 1995). Fetal hippocampal dissections included the entire structure. Adult offspring were sacrificed by decapitation 2 weeks after the final behavioral test. FX (A/P 5.2–2.7; M/L 0–3.3; D/V 9.0–5.0) and HP were immediately dissected (Paxinos and Watson, 1997). The left half of each structure was placed on dry ice and the right half was placed into RNA*later*. Tissues were stored at -80°C until use.

### RNA ISOLATION AND SEMI-QUANTITATIVE RT-PCR

Total RNA extraction was performed using Trizol reagent (Life Technologies, Gaithersburg, MD, USA) according to the manufacturer’s protocol. Genomic DNA was removed using the TURBO DNA-*free* kit (Applied Biosystems, Foster City, CA, USA). DNased RNA (1 µg) was reverse transcribed using the Promega ImPromII Reverse Transcription kit (Promega, Madison, WI, USA) and RH primers. Final product was resuspended to 100 µl as per manufacturer’s instructions. For strand-specific RT-PCR, DNased RNA (0.3 µg) was reverse transcribed using *rDio3* sense-strand (F) or OS-specific (R) primers (0.6 nM). Two microliters of final product (10 µl) was used directly in subsequent PCR amplification procedures, with addition of 0.2 nM of additional RT primer and 0.5 nM of respective Forward or Reverse primer for second strand syn-thesis and amplification. Controls included reactions without reverse transcriptase (RT^-^) or template. For semi-quantitative analysis, RH-primed total RNA samples were amplified to obtain signal within the exponential phase of the PCR reaction as follows: *rDio3os*: 60°C annealing temperature, 37 cycles; *beta-actin*: 1:4 dilution of cDNA, 55°C annealing temperature, 29 cycles, and analyzed by gel electrophoresis followed by digital imaging (Kodak Gel Logic 200, Rochester, NY, USA) and relative densitometry (Adobe Photoshop 12.0.1, San Jose, CA, USA). *rDio3os* expres-sion signals were normalized to beta-actin. Primer pairs: *rDio3os* “X”: F, 5′-CTTGGAGGGCCTGGCATTAAC; R, 5′-AAGACACT-GGCACTACTGGC; “Y”: F, 5′-AACTTTCTCGACCAGAAACC-GC; R, 5′-TAGTATAGGAGTCCGATGGC; “Z”: F, 5′-AAGCTGG-TTAAGGGTGGAGC; F*, 5′-TTGCAACTTGAGCCCTGAGGG; R, 5′-TACACCATTGCCACCACCGACTGC; *beta-actin*: F, 5′- GCTCCTCCTGAGCGCAAGTA; R, 5′-CTCCTGCTTGCTGATCCACAT.

### *rDio3os* CHARACTERIZATION AND SEQUENCING

Putative *rDio3os* sequence was identified through BLAST (http://blast.ncbi.nlm.nih.gov/Blast.cgi)> alignment of the EST BI274690 with rat chromosome 6 genomic contig reference assembly NW_047772.1 and mouse chromosome 12 reference assembly NC_000078.5. PCR primers for amplification of putative *rDio3os* transcript were designed manually based on mouse *Dio3os* exons in splice variant ESTs AY077459 and AY238181. To identify 5′ and 3′ transcript ends, RACE was performed using the Invitrogen GeneRacer kit (Life Technologies, Grand Island, NY, USA). PCR amplified products and *rDio3os* splice variants were extracted from agarose gels, purified and sequenced using the Children’s Hospital of Chicago Research Center (CHCRC) Core Facility (Chicago, IL, USA) or the Northwestern University Feinberg School of Medicine (FSM) Genetics and Genomics Core Facility (Chicago, IL USA). For SNP identification, BN, SD, and S × B genomic DNA was amplified and sequenced across the region (156500–160800 bp). Reported analyses are using T/A (SD/BN) at 158338 bp. For allele-specific expression analysis, primers flanking the SNP(s) between SD and BN rat strains were used for amplification of cDNA derived from RH-primed RT-PCR or strand-specific RT-PCR (above), with a second round of nested PCR as necessary to generate sufficient product for sequencing. Ratio of allele-specific transcripts were determined by direct measurement of sequence traces in both forward and reverse directions, normalized to genomic DNA allelic ratio (50:50). Additional primers used for nested PCR and sequencing are available upon request.

## Conflict of Interest Statement

The authors declare that the research was conducted in the absence of any commercial or financial relationships that could be construed as a potential conflict of interest.
